# Disease gene identification by random walk on multigraphs merging heterogeneous genomic and phenotype data

**DOI:** 10.1186/1471-2164-13-S7-S27

**Published:** 2012-12-07

**Authors:** Yongjin Li, Jinyan Li

**Affiliations:** 1 Center for Systems Biology, University of Texas at Dallas, USA; 2 Advanced Analytics Institute, Faculty of Engineering and IT, University of Technology, Sydney, Australia

## Abstract

**Background:**

High throughput experiments resulted in many genomic datasets and hundreds of candidate disease genes. To discover the real disease genes from a set of candidate genes, computational methods have been proposed and worked on various types of genomic data sources. As a single source of genomic data is prone of bias, incompleteness and noise, integration of different genomic data sources is highly demanded to accomplish reliable disease gene identification.

**Results:**

In contrast to the commonly adapted data integration approach which integrates separate lists of candidate genes derived from the each single data sources, we merge various genomic networks into a *multigraph *which is capable of connecting multiple edges between a pair of nodes. This novel approach provides a data platform with strong noise tolerance to prioritize the disease genes. A new idea of random walk is then developed to work on multigraphs using a modified step to calculate the transition matrix. Our method is further enhanced to deal with heterogeneous data types by allowing cross-walk between phenotype and gene networks. Compared on benchmark datasets, our method is shown to be more accurate than the state-of-the-art methods in disease gene identification. We also conducted a case study to identify disease genes for Insulin-Dependent Diabetes Mellitus. Some of the newly identified disease genes are supported by recently published literature.

**Conclusions:**

The proposed RWRM (Random Walk with Restart on Multigraphs) model and CHN (Complex Heterogeneous Network) model are effective in data integration for candidate gene prioritization.

## Background

Reliable identification of disease genes is an important task in biomedical research useful to find out the mechanism of a disease and to reveal therapeutic targets. Family based genetic linkage analysis has been widely conducted to determine regions in the chromosomes of a genome which have large genetic effects on a disease [1]. Each *susceptible region *in the chromosomes is called a susceptible locus which may cover dozens even hundreds of genes. Those genes in a susceptible locus are candidate disease genes which can be further narrowed down to the real disease genes by computational or experimental experiments. At the Online Mendelian Inheritance in Man (OMIM) database [2] which stores the latest data obtained by linkage analysis, there are still thousands of disease loci in which the real disease-causing genes have not been identified. Sophisticated computational algorithms have been recently proposed to prioritize those candidate genes [3-7] to deal with this problem. However, most of the algorithms are based on single data source. As a single data source is prone of bias, incompleteness and noise, integration of various genomic data sources is highly demanded for reliable prioritization of a set of candidate genes. The top ranked candidate genes are then most likely to be the real disease genes.

A commonly adapted data integration approach is to integrate separate lists of candidate genes derived from the each single data sources. A notable example is ENDEAVOUR [8], by which nine data sources were handled including sequence data, gene annotation data, etc. It was implemented in a rank aggregation based integration (RABI) framework consisting of two stages. In the first stage, a rank list of candidate genes is determined according to their similarity to known disease genes based on each data source. Subsequently, these rank lists are integrated into one rank list by using N-dimensional order statistics (NDOS) [9]. In an earlier work [10], we improved the performance of ENDEAVOUR by using a random walk with restart (RWR) in the first stage as the ranking algorithm, and using a discounted rating system (DRS) in the second stage to combine the ranked lists.

Merging separate lists of candidate disease genes derived from single data sources with bias and noise can inflate the uncertainties in the data and may propagate into the final ranking. To address this problem, it's better to eliminate the bias and noise by merging the single data sources into an integrated data source, and then to prioritize a set of candidate genes. This work proposes a novel integration method to merge various genomic networks into a *multigraph *which is capable of connecting multiple edges between a pair of nodes. We then operate a random walk on the multigraph to find disease genes. Many random walk models have been introduced to solve different kinds of problems in bioinformatics recently. For example, Köhler et al. [11] used the RWR algorithm to prioritize candidate genes. Macropol et al. [12] proposed a repeated random walks algorithm to predict protein complex from the PPI network. Nibbe et al. [13] used random walk models to identify disease-relevant subnetworks from the PPI network, and studied a crosstalk between them. However, none of these models can work on multigraphs as the multiple edges between a pair of nodes complicates the calculation of transition probabilities.

In this work, we first construct separate gene networks corresponding to different data sources, and then merge these networks into a single network defined by a multigraph. When our random walk algorithm runs on the merged network, the transition probability is proposed to be calculated as the expected value of the transition probabilities from the multiple networks. Our algorithm was compared with four RABI models [8,10]. On a benchmark data set covering 36 genetic diseases [11], our proposed algorithm achieved AUC value of 89.4%, much higher than the four RABI models. Our method is named RWRM (Random Walk with Restart on Multigraphs).

This work is further deepened by additionally considering phenotype data. It is widely understood that phenotype information can be used to improve the discovery of disease genes [14-16], because similar disease phenotypes are caused by mutation in functionally similar genes [17]. We do not integrate the phenotype data into the multigraph gene network. Instead, it is connected, as a subgraph, to the multigraph gene network. So, the traversals of random walk are sometimes within the multigraph gene network, sometimes within the phenotype network, and sometimes cross between these two subgraphs. We propose a Complex Heterogeneous Network (CHN) model to guide the random walk. Four genomic data sources used in this work are a PPI network and three ontologies: Biological Process (BP), Cellular Component (CC), and Molecular Function (MF). We also used the latest OMIM data as the benchmark data containing 3,871 Phenotype-Gene Relationships (PGRs) to parter with the genomic networks. We successfully identified 2,105 of the PGRs, whereas a NDOS-based [9] or a DRS-based [10] method could identify only 2,008 or 2,048 number of relationships respectively. This demonstrate that a better performance by our CHN model can be achieved. We also conduct a case study to show the excellent performance of the proposed CHN model by discovering disease genes for Insulin-Dependent Diabetes Mellitus (IDDM).

## Datasets

This section describes the datasets used by this work, including two datasets of disease genes, a phenotype network, a PPI network and functional similarity networks derived from gene ontology (GO) [18].

### Disease genes

Two benchmark datasets of disease genes are involved. The first one is obtained from Köhler et al.'s work [11]. It is constructed by processing all entries in the OMIM database [2] in which thousands of OMIM phenotypes are categorized into 110 diseases. The largest one contains 47 genes and the smallest contains only three genes. This study is focused on 36 diseases each related to at least 6 genes. The second benchmark dataset contains 3,871 PGRs obtained from the latest version of OMIM as described in detail in the following section.

### The phenotype network and phenotype-gene net-work

Every phenotype entry is defined as an MIM record, a text description of the disease phenotype. We excluded those records with the prefix '∗' and '^', because the prefix '∗' refers to an arbitrary record of disease gene, and '^' refers to an obsoleted record. We obtained 6,708 phenotypes in total. Then we calculated the similarity between phenotypes, based on the co-occurrence of key words in the Medical Subject Headings vocabulary (MeSH) [19]. This was done as follows. Every disease phenotype is first converted into a numerical vector, where each element denotes the frequency of a key word in the description of the disease phenotype. Then the similarity between two phenotypes is measured by the correlation between the two vectors. We used a text mining PERL package MimMiner [20] to calculate the similarity between every pair of phenotypes and obtained a phenotype similarity matrix.

**Figure 1:**
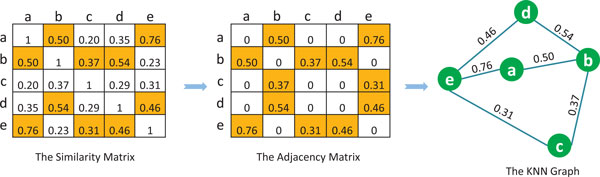
**The procedure of generating KNN graph with *K *= 2**. Each column of the similarity matrix represents the similarity between one data point and others, in which two shaded units are the 2 nearest neighbors of one data point. The adjacency matrix is derived from the similarity matrix.

With the phenotype similarity matrix, we constructed a KNN graph, i.e., phenotype network, in which each phenotype is represented as a node. We use Figure 1 to illustrate the construction steps for a KNN graph. We start from the similarity matrix of five nodes (the a, b, c, d and e nodes). For each node, we find its *K *most similar nodes. In this example, *K *is set as 2. As shown in the first column of the similarity matrix, for node a, two most similar data points are b and e. For the second column (node b), the most similar nodes are a and d. Then the KNN graph is constructed by connecting the nodes with its two most similar nodes and each edge is labeled with a weight equal to the similarity score. In this example, node b has three neighbors, because it is within two nearest neighbors of three nodes a, c and d. The same situation can be found for node e. Different phenotype networks can be constructed with different *K *sizes, and we studied the impact of *K *size on the results.

PGRs were extracted from the OMIM database [2], using BioMart [21]http://www.biomart.org/biomart/martview. The PGRs can be viewed as a bipartite network, i.e., a phenotype-gene network, in which one partite of nodes are the genes and the other partite are the phenotypes, and edges are the PGRs. This phenotype-gene network can be used as a bridge to construct a heterogeneous network as explained in a later section.

### Protein-protein interaction (PPI) network

The PPI data were derived from Human Protein Reference Database (HPRD) [22]. HPRD contains manually curated scientific information pertaining to the biology of most human proteins. All the interactions in HPRD are extracted manually from literature by expert biologists who read, interpret and analyze the published data. We excluded self-interactions. In total, there are 36,619 unique interactions between 9,474 proteins. The interaction data is used to construct a network, in which proteins and interactions are represented by nodes and edges, respectively.

### Gene Ontology and gene functional similarity net-work

Gene Ontology provides a controlled vocabulary to describe gene and gene product attributes [18]. It is comprised of three independently annotated subontologies: Biological Process (BP), Cellular Components (CC) and Molecular Function (MF).

The functional similarity between two genes can be measured by the semantic similarity between their GO annotation terms [23-26]. In our research work, the similarity between two genes was measured by their overlap annotation terms [26], because of its computational efficiency. Since there are three independent sub-ontologies, the functional similarity is defined considering three different aspects. We calculate three similarity matrices, each corresponding to a sub-ontology, then we construct the corresponding KNN graph, as illustrated in Figure 1. Specifically in this study, *K *is set as 5. (Other K's are also investigated and compared.) The obtained graphs are named BP network, CC network and MF network, respectively.

## Methods

### Random walk with restart

Random walk with restart (RWR) is a ranking algorithm, which has been used for candidate gene prioritization in the past [10,11]. Let $A_{\left(N_{g}\;\times \;N_{g}\right)}$ be the adjacency matrix of a gene network with ${\left(N\right)}_{\}}$ number of nodes and an edge set *E*. Based on the topology of the gene network, the transition matrix $M_{\left(N_{g}\times \;N_{g}\right)}$ is calculated, in which *M_i, j _*denotes its (*i, j*)-th element, representing the probability of transition from node *i *to node *j*. The calculation of *M_i, j _*is given by

(1) $$M_{i,j}=\{\begin{array}{cc} A_{i,j}/d(i) & \text{if}\;e(i,j)\in \;E\\ 0, & \text{otherwise,} \end{array}$$

where *d*(*i*) is the sum of the *i*-th column in *A*. The RWR algorithm updates the probability vectors by

(2) $$p_{s\text{ + 1}}=\left(1-\gamma \right)M^{T}p_{s}+\gamma p_{0},$$

where *M^T ^*is the transpose matrix of *M *and **p**_0 _is the initial probability vector. In this work, the initial probability vector **p**_0 _is set such that equal probabilities are assigned to all the source nodes with the sum of the probabilities equal to 1. The probability *p*_∞ _(*i*) is the probability of finding the random walker at node *g_i _*in the steady state. It gives a measure of proximity between *g_i _*and source nodes. If *p*_∞ _(*i*) >*p*_∞ _(*j*), then node *g_i _*is more proximate to source nodes than node *g_j _*does.

### Random walk with restart on multigraph gene net-works

We extend the RWR algorithm to operate on multigraph gene networks and propose an RWRM (Random Walk with Restart for Multigraphs) algorithm to prioritize a set of candidate genes.

**Figure 2:**
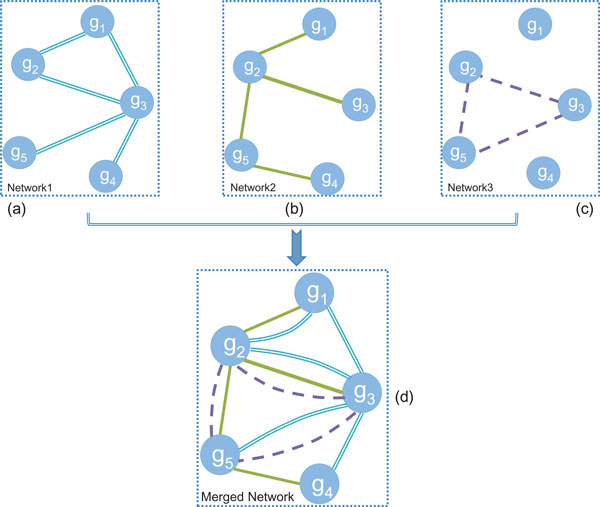
**Construction of a multigraph by merging**. Three kinds of links denote three kinds of relationships obtained from different data sources.

Figure 2 (a), (b) and 2(c) present three different networks with the same set of nodes {*g*_1_, *g*_2_, *g*_3_, *g*_4_, *g*_5_}, constructed by using relationships from three different data sources. The merged network as shown in Figure 2(d) is a multigraph, sharing the same set of nodes and containing all of the edges from the three separate networks. Between any pair of nodes in the merged network, its multiple edges are all and only inherited from the same pair of nodes of the three networks. For example, there are two links between node *g*_1 _and node *g*_2_, which are exactly from Network1 and Network2. The random walker can move from *g*_1 _to *g*_2 _via any of the two links. The transition probability from node *g*_1 _to node *g*_2 _on the merged network is calculated as the expected value of the transition probabilities corresponding to all of the links between node *g*_1 _and node *g*_2_.

Considering node *g_i _*in the merged network, let *N_i _*be the number of networks to which node *g_i _*is associated. For example, in Figure 2(d), *N*_1 _= 2, *N*_2 _= 3 and *N*_3 _= 3. For each of the *N_i _*networks, we calculated the transition matrix using Eq.1. Let $M_{\left(N_{g}\;\times \;N_{g}\right)}^{\left(k\right)}$ be the transition matrix of the network *k *(*k *= 1, 2, ..., *N*) and $M_{i,j}^{\left(k\right)}$ is the (*i, j*)-th element of the matrix. The transition probability from node *g_i _*to node *g_j _*is calculated as

(3) $$M_{i,j}=\sum_{k=1}^{N_{i}}q^{\left(k\right)}M_{i,j}^{\left(k\right)}\;\;\;(1\leq i,j\leq N_{g})$$

where *q*^(*k*) ^is the probability of selecting the *k*-th network.

We assume that the random walker chooses any network with equal probability $\left(q^{\left(k\right)}=\frac{1}{N_{i}}\right)$ As an example, node *g*_1 _in Figure 2 is involved in two networks Network1 and Network2, i.e., *N*_1 _= 2. The transition probability from node *g*_1 _to node *g*_2 _is then calculated as $p\left(g_{1}\to g_{2}\right)=\frac{1}{2}\;\times \;\frac{1}{2}\;+\;\frac{1}{2}\;\times \;1=0.75$. Similarly The transition matrix M for the merged network in $p\left(g_{2}\to g_{3}\right)=\frac{1}{3}\;\times \;\frac{1}{2}\;+\;\frac{1}{3}\;\times \frac{1}{3}\;\times \;\frac{1}{2}\;=0.44$. Figure 2 is

$$\begin{array}{c} \;\begin{array}{ccccccc} \;\; & g_{1} & \;g_{2} & \;g_{3} & \;g_{4} & \;g_{5} & \end{array}\\ \begin{array}{c} g_{1}\\ g_{2}\\ g_{3}\\ g_{4}\\ g_{5} \end{array}\left(\begin{array}{ccccc} 0 & 0.75 & 0.25 & 0 & 0\\ 0.28 & 0 & 0.44 & 0.28 & 0\\ 0.08 & 0.58 & 0 & 0.08 & 0.26\\ 0 & 0 & 0.5 & 0 & 0.5\\ 0 & 0.33 & 0.5 & 0.17 & 0 \end{array}\right) \end{array}$$

As another example, suppose we want to find the most proximate node with *g*_4 _in Figure 2(d). Then we set *g*_4 _as the source node to run the RWRM algorithm. The initial probability is *p*_0 _= [ 0 0 0 1 0 ]*^T^*. After running the RWRM algorithm, we get the stationary probability *p*_∞ _= [ 0.01 0.04 0.13 0.71 0.06 ]*^T^*. Node *g*_3 _has the highest stationary probability, therefore it is found to be the most proximate to the source node *g*_4_. If *g*_4 _is the known disease gene, we may infer that gene *g*_3 _is possibly a disease gene.

### Random walk on heterogeneous networks

**Figure 3:**
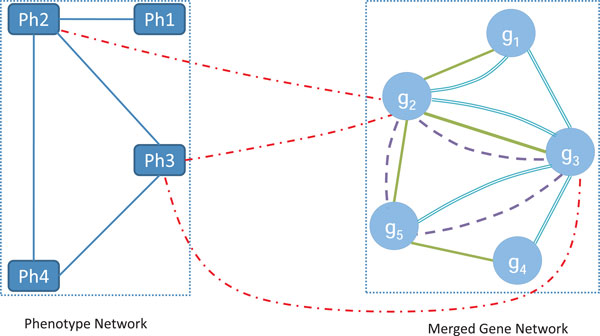
**A Complex Heterogenous Network (CHN) is constructed by connecting between a phenotype network and a multigraph gene network**. Nodes between these two types of networks are called bridging nodes. The other nodes are called internal nodes.

We propose a Complex Heterogeneous Network (CHN) model to combine a multigraph gene network and phenotype information. As illustrated in Figure 3, a CHN is constructed by connecting a phenotype network and a merged multigraph gene network, through the use of the PGRs from the OMIM database. Those nodes of a CHN connecting the merged gene network and the phenotype network are named *bridging nodes*, and the other nodes are called *internal nodes*. For example in Figure 3, nodes *Ph*2, *Ph*3, *g*_2_, and *g*_3 _are bridging nodes, the other nodes are internal nodes.

When the random walker moves to a bridging node, it may jump to the other subnetwork with a probability *λ *or move back to the other nodes in its home subnetwork with the probability 1 - *λ*. The parameter *λ *is called *jumping probability*, which regulates the reinforcement between the multigraph gene network and phenotype network. The transition from the gene network to the phenotype network (vice verse) is called an inter-subnetwork transition. If the transition is within the gene network or within the phenotype network, it is called an intrasubnetwork transition. The transition matrix M for our CHN model is given by

(4) $$M=\left[\begin{array}{ccc} {\left(M_{G}\right)}_{N_{g}\times \;N_{g}} & & {\left(M_{GP}\right)}_{N_{g}\times \;N_{Ph}}\\ {\left(M_{PG}\right)}_{N_{Ph}\;\times \;N_{g}} & & {\left(M_{P}\right)}_{N_{Ph}\;\times \;N_{Ph}} \end{array}\right]$$

where *M_G _*is the transition matrix of the gene network; *M_P _*is the transition matrix of the phenotype network; *M_GP _*is the transition matrix from the gene network to the phenotype network; and *M_PG _*is the transition matrix from the phenotype network to the gene network. The intra-subnetwork transition matrix, *M_P _*and *M_G_*, are calculated by using Eq.1 and Eq.3, respectively. The transition probability from a bridging node to the other nodes in the same subnetwork is modified by multiplying 1 - *λ*. For example, using Eq.3, the transition probability from node *g*_2 _to *g*_3 _is calculated as *p*(*g*_2 _→ *g*_3_) = 0.44. However in the CHN model, it becomes 0.44 × (1 - *λ*). If *λ *= 0.5, *p*(*g_i _*→ *g_j_*) = 0.44 × 0.5 = 0.22. The transition probability from an internal node remains unchanged. For example, the transition probability from the internal node *g*_1 _to *g*_2 _is 0.75 in both the merged gene network and the CHN model.

The inter-subnetwork transition probability from node *g_i _*to node *Ph_j_, p*(*g_i _*→ *Ph_j_*) is given by

(5) $${\left(M_{GP}\right)}_{i,j}=\left\{\begin{array}{cc} \frac{\lambda B_{i,j}}{\sum_{j\;=\;1}^{N_{Ph}}B_{i,j}} & \text{if}\;\sum_{j}B_{i\;j}\neq 0\\ 0, & \text{otherwise} \end{array}\right)$$

where $B_{N_{\text{g}}\times N_{Ph}}$ is the adjacency matrix of the gene-phenotype network. Similarly, the transition probability from node *Ph_i _*to node *g_j_, p*(*Ph_i _*→ *g_j_*), is given by

(6) $${\left(M_{PG}\right)}_{i,j}=\left\{\begin{array}{cc} \frac{\lambda B_{i,j}}{\sum_{j\;=\;1}^{N_{g}}B_{j,i}} & \text{if}\;\sum_{j}B_{j\;i}\neq 0\\ 0, & \text{otherwise} \end{array}\right)$$

Let **u**_0 _and **v**_0 _be the initial probability vectors for gene network and phenotype network, respectively. Then the initial probability vector for the CHN model is represented as

(7) $$p_{0}=\left[\begin{array}{c} (1-\eta )u_{0}\\ \eta v_{\text{0}} \end{array}\right],\;(0<\eta <1)$$

The parameter *η *is used to weight the importance of each sub-network. If *η *is 0.5, two sub-networks are equally weighted. If *η *is above 0.5, the random walker prefers to return to the phenotype source node, therefore the phenotype information is assigned with more importance.

The initial probability of gene network **u**_0 _is assigned such that equal probabilities are set to all the source nodes in the gene network, with the sum of the probabilities equal to 1. The initial probability of phenotype network **v**_0 _is given similarly. Suppose *g*_1_, *g*_4 _and *g*_5 _in Figure 3 are candidate genes for phenotype *Ph*3. To find the real disease gene, we use *g*_2_, *g*_3 _and *Ph*3 as source nodes to run the above random walk algorithm. In this case, $u_{0}\text{ = }{\left[0\;\;\frac{1}{2}\;\;\frac{1}{2}\;\;0\;\;0\right]}^{T}\;\text{and}\;\;v_{0}={\left[0\;\;0\;\;1\;\;0\right]}^{T}$.

The transition matrix *M *is calculated as:

$$\begin{array}{c} \begin{array}{ccccccccc} \;\;\;\;\;\; & g_{1} & \;\;\;g_{2} & \;\;\;g_{3} & \;\;g_{4} & \;\;\;g_{5} & \;\;Ph_{1} & \;Ph_{2} & \;Ph_{3}\;\;\;\;\;Ph_{4} \end{array}\\ \begin{array}{c} g_{1}\\ g_{2}\\ g_{3}\\ g_{4}\\ g_{5}\\ Ph_{1}\\ Ph_{2}\\ Ph_{3}\\ Ph_{4} \end{array}\left(\begin{array}{cccccccccc} 0 & 0.75 & 0.25 & 0 & 0 & & 0 & 0 & 0 & 0\\ 0.14 & 0 & 0.22 & 0.14 & 0 & & 0 & 0.25 & 0.25 & 0\\ 0.04 & 0.29 & 0 & 0.04 & 0.13 & & 0 & 0 & 0.5 & 0\\ 0 & 0 & 0.5 & 0 & 0.5 & & 0 & 0 & 0 & 0\\ 0 & 0.33 & 0.5 & 0.17 & 0 & & 0 & 0 & 0 & 0\\ 0 & 0 & 0 & 0 & 0 & & 0 & 1 & 0 & 0\\ 0 & 0.5 & 0 & 0 & 0 & & 0.17 & 0 & 0.17 & 0.17\\ 0 & 0.25 & 0.25 & 0 & 0 & & 0 & 0.5 & 0 & 0.5\\ 0 & 0 & 0 & 0 & 0 & & 0 & 0.5 & 0.5 & 0 \end{array}\right) \end{array}$$

We substitute the transition matrix M and initial probability **p**_0 _into the iterative equation (Eq.2). After a number of iteration steps, a steady probability matrix

(8) $$p_{\infty }=\left[\begin{array}{c} (1-\eta )u_{\infty }\\ \eta v_{\infty } \end{array}\right]$$

can be obtained. Then genes and phenotypes can be ranked according to this steady probability **u_∞ _**and **v_∞_**, respectively. The larger the probability value is, the higher the rank position.

### Rank aggregation based integration: literature work for comparison

Our proposed ranking methods are compared with various rank aggregation based integration (RABI) methods for performance evaluation. RABI methods prioritize a set of candidate genes derived from individual data source, and then combine these rank lists into a single list. A RABI has two steps: ranking and rank aggregation. For the first step, we considered using one-class support vector machine (1CSVM) [27,28], RWR [11] and RWRH [16]. For the second step, we considered using N-dimensional order statistics (NDOS) [8] and discounted rating system (DRS) [10]. Therefore, six RABI models were used for comparison in this work, namely 1CSVM+NDOS, 1CSVM+DRS, RWR+NDOS, RWR+DRS, RWRH+NDOS and RWRH+DRS. The first four models are compared with our RWRM model, which integrates multiple gene networks. The last two models are compared with the CHN model, which integrates multiple gene networks and the phenotype network.

The 1CSVM method requires a kernel representation of the data. We used the diffusion kernel [29,30], with the diffusion parameter set as 0.25. In RWR and RWRH, the *γ *value was set as 0.7. It has been shown that the performance of RWR algorithm is stable with *λ *ranging from 0.6 to 0.9 [10]. In the DRS algorithm, there is one parameter, the number of ratings, which has little impact on the results [10]. In this work, candidate genes were classified into five ratings. There is no parameter in the NDOS algorithm.

## Experiments and results

This section reports a comparative performance of our new methods on two benchmark datasets. As mentioned, the first dataset consists of 36 diseases collected by [11]. The second dataset is the set of the 3,871 PGRs obtained from OMIM [2]. Our proposed RWRM and CHN models are compared with the six RABI models by using the ROC and other criteria. As a complicated case study, we also use the proposed CHN model to discover previously unknown disease genes for Insulin-Dependent Diabetes Mellitus (IDDM).

### Performance of the RWRM model on 36 diseases

As explained in the Dataset section, the PPI data is collected from HPRD [22]. Together with the three gene networks from the ontology BP, CC and MF, the PPI network is merged into a single network, containing 307,823 interactions of 14,529 genes. Table 1 shows the sizes of these networks.

**Table 1:** Overview of Four Gene Networks and the Merged Gene Network

Data Source	Nodes	Edges
PPI Network	9,474	36,619
GO-BP	9,740	74,184
GO-CC	7,400	64,709
GO-MF	10,661	132,311
Merged Network	14,529	307,823

**Figure 4:**
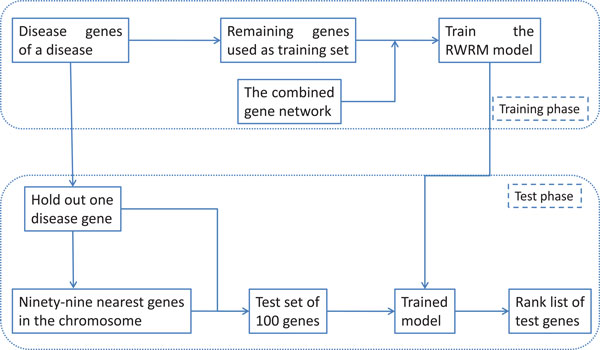
**The procedure of leave one out cross validation**.

We applied the proposed RWRM algorithm on the benchmark dataset of 36 diseases. The performance was evaluated using leave one out cross validation which is outlined in Figure 4. For a given disease, suppose there are $N_{dg}$ disease genes. We hold out one gene from the set of disease genes. The rest $N_{dg}-1$ genes are used as source nodes in the RWRM algorithm. The held-out disease gene along with the 99 nearest genes in the chromosome were used as test genes. They were ranked by the RWRM algorithm with *γ *set as 0.7. Then, for each disease gene, we obtained a rank list of 100 test genes. Then, another disease gene of the selected disease was held-out. As this procedure repeated, all of the disease genes were prioritized. So, we had $N_{dg}$ rank lists, each for a disease gene. Since there were total 497 disease genes for the 36 diseases, we obtained 497 rank lists in total. By using the $N_{dg}$ rank lists, the ROC curve was plotted and the disease specific AUC values were calculated, namely 36 AUC values calculated corresponding to the 36 diseases. Using the entire 497 rank lists, we calculated the overall AUC value.

**Figure 5:**
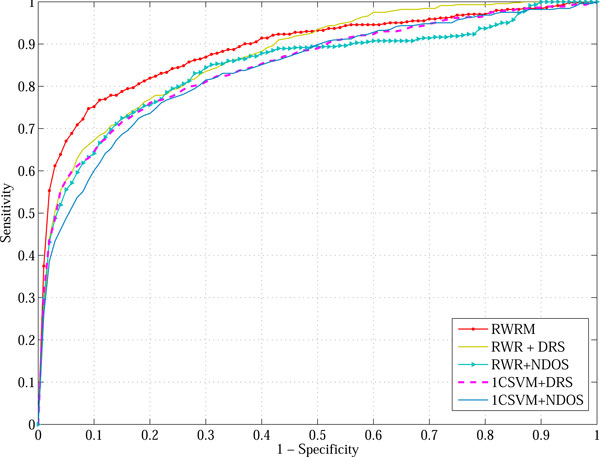
**ROC curves of RWRM and four RABI models, on the benchmark dataset of 36 diseases**.

In comparison to the four RABI methods RWR+DRS, RWR+NDOS, 1CSVM+NDOS and 1CSVM+NDOS, the overall ROC curve of our RWRM is drawn in Figure 5. From the left part of this figure, it can be seen that the curve corresponding to RWRM is above all of the other four. It indicates that the RWRM algorithm ranked more number of disease genes at the top than the RABI models. The overall AUC value of RWRM is 89.4%, higher than all of the RABI models (Table 2). From Figure 5 and Table 2, it can be also seen that the RWR+DRS model performed better than the other three RABI models. Therefore, we chose to compare the performance between RWRM and RWR+DRS on the individual diseases. We performed Wilcoxon signed-rank test [31] on the AUC values obtained by RWRM and RWR+DRS. Wilcoxon signed-rank test is a non-parametric alternative to the pair-wise t-test. It can be used to test the difference between pair-wised samples, when the population cannot be assumed to be normally distributed. The null hypothesis is that there is no significant difference between the results of two methods. We found that AUC values obtained by the RWRM algorithm are significantly higher than RWR+DRS (*p *= 0.047) on the benchmark data set of 36 diseases.

**Table 2:** Performance of five different integration models on 36 diseases in terms of overall AUC values (%)

Integration Models	Overall AUC (%)
RWRM	89.4
RWR+DRS	87.7
RWR+NDOS	85.1
1CSVM+DRS	85.4
1CSVM+NDOS	84.4

### The performance of the CHN model

The PPI network and the three ontology networks BP, CC and MF were used to construct the multigraph part of the heterogeneous network which was then finalized by using the PGRs to connect to the phenotype network. The leave one out cross validation was also taken to evaluate the performance of the CHN model. In each round of cross validation, one PGR was removed from the dataset of 3,871 PGRs. The removed gene is called held-out gene and the removed disease phenotype is called target phenotype. The aim of this cross validation is to test whether or not the CHN model can successfully predict the relationship between the held-out gene and the target phenotype. We also collected 99 genes in the nearest chromosome region of the held-out disease gene, and our set of test genes constituted these 99 genes and the held-out gene.

**Figure 6:**
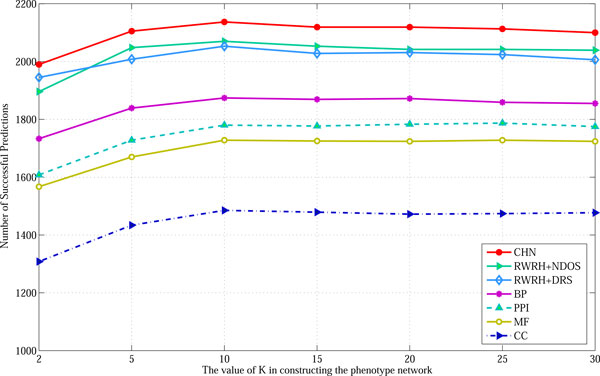
**The impact of *K *value in the KNN graph on the results**. This figure shows the performance of single networks from BP, CC, MF and PPI, constructed with various *K *values, and the performance of three integration models.

We used the CHN model to prioritize this set of test genes, in which the target phenotype and the rest of the associated disease genes were assigned as source nodes. The parameters of the CHN model were set as *γ *= 0.7, *λ *= 0.5 and *η *= 0.5. If the held-out disease gene is ranked at the top, it is taken as a successful prediction. The number of successful predictions is used as the performance measure. We considered a series of *K *sizes to construct the KNN graph of phenotype, ranging from 2 to 35. Results are shown in Figure 6. The number of successful predictions for the CHN model increases from 1998 to 2137, when *K *increases from 2 to 10. Then the number of successful predictions decreases slightly when *K *increases from 10 to 35. The same trend can be observed for both RABI models and RWRH models. In comparison to the two RABI models, our CHN model always achieved better results when K was varied. The performance of the two RABI models 'RWRH+DRS' and 'RWRH+NDOS', was competitive, and both of them performed better than the RWRH models which is based on individual gene networks. These results demonstrate that the bridging between the multigraph gene network and the phenotype network is important to improve the performance.

### Disease genes of insulin-dependent diabetes mellitus identified by CHN: a case study

Insulin-Dependent Diabetes Mellitus (IDDM) is commonly known as Type 1 diabetes mellitus, which is a consequence of autoimmune destruction of insulin-producing pancreatic beta cells. It is a genetically heterogeneous autoimmune disease, with multiple genes and loci involved in the pathogenesis [32]. Different susceptible loci correspond to different phenotypes (i.e., MIM IDs in the OMIM database [2]). In some susceptible loci, real disease genes have been found. But in some other loci, we only know a list of candidate genes and need to find real disease genes. Table 3 shows 14 loci associated with IDDM, where the second column shows the MIM ID of the disease phenotype, the third column is the associated locus, and the fourth column shows the number of candidate genes in the susceptible locus.

**Table 3:** Top five ranked candidate genes for each phenotype of IDDM

SN	MIM ID	Locus	Number of Candidate Genes	Top 5 Candidate Genes
1	600318	15q26	72	BLM, IGF1R, FURIN, RHCG, **NR2F2**
2	600319	11q13	263	PPME1, RELA, STIP1, GSTP1, MEN1
3	601941	18q21	88	BCL2, TXNL1, MYO5B, SMAD4, TNFRSF11A
4	600321	2q31	81	**NEUROD1**, ABCB11, WIPF1, LRP2, CCDC141
5	600883	6q25-q27	38	ESR1, PLG, TBP, VIL2, IGF2R
6	601208	14q24.3-q31	85	TSHR, FOS, EIF2B2, NEK9, PTPN21
7	601318	2q34	19	IDH1, ERBB4, PIP5K3, LANCL1, PTHR2
8	601666	6q21	51	FYN, CDC40, ATG5, CD164, TUBE1
9	603266	10q25	55	**TCF7L2**, GFRA1, VTI1A, ACSL5, **ADRA2A**
10	605598	5q31.1-q33.1	241	SPINK1, **NR3C1**, VDAC1, HSPA9, IL3
11	612520	12q24	216	SH2B3, TCF1, OAS1, PTPN11, OAS2
12	612521	6q25	70	ESR1, SYNE1, VIL2, LATS1, OPRM1
13	612622	4q27	17	CCNA2, IL2, MAD2L1, FGF2, TRPC3
14	613006	10q23.31	26	PTEN, ACTA2, PANK1, ANKRD1, MPHOSPH1

Given a phenotype, the set of candidate genes was obtained by using BioMart [21]. Then our CHN model was used to prioritize this set of candidate genes. The parameters for the CHN model were set as *γ *= 0.7, *λ *= 0.5 and *η *= 0.5. Top five ranked candidate genes are shown in the fifth column of Table 3 with a decreasing relevance from the left to the right. Some of these predictions can be affirmed with literature work.

The fifth ranked gene for MIM 600318 (Serial No. 1 in Table 3) is NR2F2 (also called COUP-TFII). It has been found to contribute to the control of insulin secretion through the complex HNF4 transcription factor network operating in chicken pancreatic beta cell [33]. This is suggestive of that the mutation studies in the human NR2F2 gene are important to understand more about IDDM.

The first ranked gene for MIM 600321 (Serial No. 4 in Table 3) is NEUROD1. A literature work by Cinek et al. [34] found a close association between NEUROD1 and IDDM in Czech children. They compared 285 children with IDDM diagnosed under the age of 15 years with 289 non-diabetic control children to confirm that NEUROD1 polymorphism Ala45Thr is associated with IDDM.

The locus of MIM 603266 (Serial No. 9 in Table 3) overlaps with the loci of type 2 diabetes (T2D). Therefore those genes in this particular region may explain the common mechanisms of both types of diabetes. The first ranked gene TCF7L2 is known as T2D causing gene and the fifth ranked gene ADRA2A is hypothesized to increase T2D risk [35]. A single-nucleotide polymorphism (SNP) in the human ADRA2A gene has been found responsible to reduced insulin secretion [35]. All these suggest that important associations of IDDM with TCF7L2 or with ADRA2A are likely to be established through further mutation analysis.

NR3C1 is known as GR (Glucocorticoid Receptor). It is ranked the second among the set of 241 candidate genes for MIM 605598 (Serial No. 10 in Table 3). Rosmond and Holm [36] performed a five-year follow-up study on 163 unrelated Swedish men for investigating 3 polymorphisms of this gene. They found a significant increase in body weight, body mass index, abdominal obesity, fasting glucose, insulin, and homeostasis model assessment over the 5-year follow-up among homozygotes for the rare BclI allele. Syed et al. [37] also reported an SNP in the human GR gene (rs2918419) which is linked with insulin resistance in men. These results indicate a high possibility that NR3C1 is likely to be associated with IDDM18.

## Conclusions and discussion

In this paper, we have proposed two random walk models applicable to merged data for candidate gene prioritization and identification of disease genes. The RWRM model is an extended version of random walk algorithms specially designed for multigraph gene networks integrating various data sources. It was compared to the state-of-the-art RABI models and improved performance has been achieved. We also combined the phenotype information with the multigraph gene networks and proposed the CHN model. The CHN model is also found to be better than the RABI models in disease gene prediction.

We are interested in several future research directions. One topic is how to assign proper weights to different data sources. In this paper, we simply give all the data sources equal importance. Biologists may give the weights to different data sources based on their expert knowledge. Another problem is that the transition probability corresponding to individual gene networks is calculated independently before combination. For better performance, the transition probability should be calculated not only based on the topology of the current network but also based on other networks. The third direction is about the case study. Some of the top-ranked genes have been affirmed with literature work. As our prediction performance is high in accuracy, it is believed that those top-ranked but un-affirmed genes are new and potentially useful research targets in the field of disease gene prioritization.

## Competing interests

The authors declare that they have no competing interests.

## Authors' contributions

YJ Li designed the algorithms and drafted the initial manuscript. JY Li provided advice related to data processing and analysis and revised the manuscript. Both authors read and approved the final manuscript.
